# Severe Headache and Deterioration of Vision in Left Eye in a Chronic Hemodialysis Patient Revealing a Brown Tumor of Sphenoid Sinus

**DOI:** 10.3390/neurolint17020022

**Published:** 2025-01-30

**Authors:** Hicham Esselmani, Abdelmohcine Aimrane, Hicham Chatoui, Omar El Hiba, Mustapha Najimi, Mohamed Merzouki

**Affiliations:** 1 Biological Engineering Laboratory, Faculty of Sciences and Techniques, Sultan Moulay Slimane University, Beni Mellal 23000, Morocco; esselmani1@yahoo.fr; 2 Anthropogenetics, Biotechnologies and Health Laboratory, Nutritional Physiopathologies, Neurosciences and Toxicology Team, Faculty of Sciences, Chouaib Doukkali University, El Jadida 24000, Morocco; aimrane.a@ucd.ac.ma (A.A.); oelhiba@gmail.com (O.E.H.); 3 Higher Institute of Nursing Professions and Health Techniques (ISPITS-M), Ministry of Health, Marrakech 40000, Morocco; chatouih@yahoo.fr; 4 Laboratory of Pediatric Hepatology and Cell Therapy, Institute of Experimental and Clinical Research, UClouvain, 1200 Brussels, Belgium; mustapha.najimi@uclouvain.be

**Keywords:** brown tumor, secondary hyperparathyroidism, sphenoid sinus, hemodialysis, hypercalcemia

## Abstract

Background/Objectives: Brown tumors are rare bone lesions associated with hyperparathyroidism, particularly secondary hyperparathyroidism (SHPT), in chronic renal failure. While brown tumors commonly affect bones rich in marrow, the involvement of the sphenoid sinus is extremely rare and can present with neurological symptoms. This study reports a case of a sphenoid sinus brown tumor in a patient on hemodialysis, highlighting its clinical presentation and diagnostic challenges. Methods: A 31-year-old woman undergoing chronic hemodialysis presented with a severe headache, diplopia, and progressive vision loss in her left eye. Laboratory tests revealed hypercalcemia, hypophosphatemia, and elevated parathyroid hormone (PTH) levels, consistent with SHPT. The diagnosis was confirmed through a clinical examination and magnetic resonance imaging (MRI). Results: The clinical examination confirmed decreased visual acuity in the left eye. The laboratory results revealed serum calcium of 15.5 mg/dL, phosphate of 1.0 mg/dL, and PTH of 2000 pg/mL, consistent with SHPT. The imaging studies identified a brown tumor in the sphenoid sinus exerting a mass effect on adjacent structures. This case underscores the rarity of brown tumors in this location, with very few similar reports in the literature. Conclusions: Although rare, brown tumors should be considered in patients with SHPT who present with neurological symptoms or cranial lesions. An early diagnosis through biochemical and imaging studies is crucial to prevent severe complications. The management involves treating the underlying hyperparathyroidism, with surgical intervention indicated in cases of neural compression.

## 1. Introduction

Brown tumors (BTs), commonly known as osteitis fibrosa cystica, are rare lytic bone lesions that are generally associated with hyperparathyroidism. While invasive in some cases, they are not potentially malignant [1,2,3]. BTs are associated with primary hyperparathyroidism (PHPT)—with a low incidence (less than 5%)—or even with secondary hyperparathyroidism (SHPT), where the incidence may reach up to 13% of cases [4]. It is worth noting that PHPT is the most common cause of hypercalcemia in the ambulatory setting [5]. With the introduction of serum calcium assessment as part of the laboratory test battery, the clinical presentation of primary PHPT has shifted from a diagnosis based on its cardinal symptoms to incidental discoveries in asymptomatic patients [6]. This may explain the increased incidence and prevalence of PHPT. The excess of parathyroid hormone (PTH) has been associated with several physiological manifestations, including an increased number and activity of osteoclasts, massive secretion of PTH, intralesional hemorrhage, and hemosiderin deposition, all of which contribute to the brownish color of the tissue. These changes can lead to asymptomatic swelling or a painful exophytic mass and may result in pathological fractures or skeletal pain, which are often described as lytic bone lesions [7]. Consequently, a BT can be confused with a carcinoma or multiple myeloma. Altered cranial bone morphology, particularly in the sphenoid sinus, can lead to neurological deficits as a primary clinical manifestation, although this occurrence is rare [8]. Diagnosing a BT in the context of HPT is primarily based on the biochemical assessment of serum calcium, phosphorus, and parathyroid hormone levels, while histology is often insufficient to confirm the diagnosis.

The incidence of BTs in hemodialysis patients has decreased due to regular biochemical monitoring and the advent of newer treatments. While a parathyroid adenoma is the most common etiology (85%), parathyroid hyperplasia and parathyroid carcinoma represent 14% and ≤1% of the cases, respectively [9]. Unlike adenomas, parathyroid carcinomas present equally between the two genders and are associated with higher calcium levels (≥14.0 mg/dL), neck mass (35%), bone disease (50%), renal disease (30%), and pancreatitis (15%) [10,11].

The pathogenesis of brown tumors is closely linked to the dysregulation of calcium and phosphate metabolism. In primary hyperparathyroidism, the overproduction of PTH leads to increased bone resorption, which can result in the formation of cystic lesions filled with fibrous tissue and hemosiderin. In secondary hyperparathyroidism, the pathophysiology is more complex, involving chronic kidney disease, vitamin D deficiency, and phosphate retention, all of which contribute to the development of these lesions. The rarity of brown tumors in developed countries is largely due to the early detection and treatment of hyperparathyroidism, but in regions with limited access to healthcare, these lesions remain a significant clinical challenge.

Herein, we report a rare case of a brown tumor located in the sphenoid sinus of a patient on chronic hemodialysis with SHPT, who presented with a severe headache and deterioration of vision in the left eye.

## 2. Observation

A 31-year-old woman was diagnosed in 2016 with end-stage renal failure of an unknown cause. Subsequently, she was maintained on chronic hemodialysis, undergoing two sessions per week, and was prescribed antihypertensive medication and a calcium supplement.

She presented for the evaluation and management of a 1-month history of severe headache, intermittent nausea, diplopia, and progressive vision loss in her left eye. On physical examination, her blood pressure was 160/110 mmHg and her pulse was 88/min and rhythmic. The clinical examination revealed decreased visual acuity in the left eye (1/10) with normal visual fields. A fundoscopic examination showed no abnormalities.

The patient’s history of chronic kidney disease and long-term hemodialysis placed her at high risk for secondary hyperparathyroidism and its complications. Her symptoms of headache, nausea, and visual disturbances were highly suggestive of a space-occupying lesion in the cranial region, prompting further investigation.

Laboratory tests revealed a normochromic normocytic anemia (8 g/dL), serum calcium of 15.5 mg/dL (8.2–10.2), phosphate of 1.0 mg/dL (2.3–4.7), albumin of 3.9 mg/dL (3.5–5.0), parathyroid hormone (PTH) of 2000 pg/mL (18–50), alkaline phosphatase of 262 IU/L (50–120), and 25-hydroxyvitamin D of 18 ng/mL (30–70). The rheumatoid factor, C-reactive protein, anti-cyclic citrullinated peptide antibody, thyroid function tests, and other hepatic function tests were within normal limits.

The biochemical findings were consistent with severe hyperparathyroidism, characterized by markedly elevated PTH levels, hypercalcemia, and hypophosphatemia.

**Figure 1:**
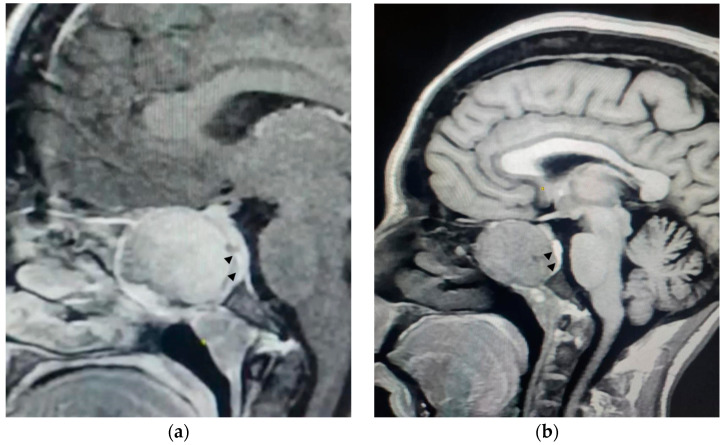
(**a**) A T1-weighted sagittal MRI with a gadolinium injection confirms the tissue nature of the mass and its expansion against the bony cortex (arrowheads). (**b**) A T1-weighted sagittal MRI without a gadolinium injection shows a lesion centered on the sphenoid sinus, expanding the bony cortex of the sinus, displacing the flattened pituitary gland posteriorly, filling the opto-chiasmatic cisterns superiorly, and making intimate contact with the optic nerve.

Magnetic resonance imaging (MRI) of the brain demonstrated a lesion centered in the sphenoid sinus, expanding the bony cortex of the sinus, which displaces the flattened pituitary gland posteriorly (Figure 1). Ultrasonography of the parathyroid glands revealed no abnormality. These findings were considered compatible with a sphenoid sinus brown tumor secondary to hyperparathyroidism.

The imaging findings were critical in confirming the diagnosis of a brown tumor. The lesion’s location in the sphenoid sinus and its impact on surrounding structures, such as the pituitary gland and optic nerve, explained the patient’s visual symptoms and highlighted the need for prompt intervention.

## 3. Discussion

BTs are benign, tumor-like bone lesions that were first described by Recklinghausen in 1891. They may affect various bone sites, such as the ribs, clavicles, pelvic bones, and mandible [7]. A body of evidence supports the involvement of the parathyroid gland in the pathogenesis of BTs, with uncontrolled HPT being the direct etiology of the disease [9]. Epidemiological studies have estimated the incidence of BTs in HPT to range from 1% to 3% in cases of PHPT and up to 13% in SHPT [4]. Additionally, the incidence of BTs appears to be more common in developing countries due to the limited access to medical care and screening programs. However, with the advancements in screening techniques and laboratory biochemical tests, PHPT can now be diagnosed even before the onset of a BT. This has led to the dissociation of BTs from PHPT as an early manifestation, occurring before the onset of systemic manifestations.

BTs are named for their brownish color, which is due to hemorrhages and the accumulation of hemosiderin within the vascularized fibrous tissue. BTs are not true neoplasms; rather, they are benign reactive lesions caused by disturbed bone remodeling. They occur due to chronically elevated levels of PTH and present as vascularized fibrous tissue containing hemorrhages and hemosiderin deposits.

A study by Carsote et al. reported that brown tumors occur in approximately 2% of patients with PHPT, highlighting the importance of early detection [11]. Due to improved screening techniques, hyperparathyroidism can now be diagnosed at earlier stages. This early detection and treatment have led to a decrease in the incidence of brown tumors, which are a classic manifestation of hyperparathyroidism [4].

Primary hyperparathyroidism (PHPT) is an endocrine disease that results from the excessive production of PTH, leading to an imbalance between the osteoblast and osteoclast activity. This imbalance results in bone resorption and the formation of brown tumors [11]. SHPT typically arises from chronic renal failure, where impaired renal function leads to decreased calcium levels. This triggers compensatory increases in PTH production, causing further bone resorption and making individuals more susceptible to brown tumors [4].

The pathogenesis of SHPT involves impaired renal function, which reduces calcitriol synthesis, the active form of vitamin D, crucial for calcium homeostasis. In response to the decreased calcium, serum phosphate levels rise, further stimulating the PTH production. This cycle of increased PTH leads to bone resorption and a predisposition to brown tumors [12]. The most common affected sites of a brown tumor are bones with rich marrow content, such the mandible, maxilla, clavicle, ribs, and pelvic bones. Consequently, the occurrence of brown tumors in the sphenoid sinus is extremely rare [8]. Our case of a brown tumor located in the sphenoid sinus is rare but not unprecedented. A review of published cases in the literature reveals that only eight cases of sphenoid sinus brown tumors (BTs) have been documented to date [13,14,15,16,17,18,19]. Among these, three cases were associated with secondary hyperparathyroidism (SHPT), similar to our study. For example, a case reported by Takeshita et al. involved a patient with SHPT who presented with a sphenoid sinus BT, confirmed by CT and MRI imaging [13]. The lesion exhibited the characteristic features, including bony expansion and compression of adjacent structures, which aligns with the findings in our case.

Another notable case was described by Schweitzer et al., in which a patient with primary hyperparathyroidism (PHPT) presented with a sphenoid sinus BT causing hypercalcemia and blindness [14]. This case underscores the potential severity of neurological complications associated with sphenoid sinus involvement, similar to the visual disturbances observed in our patient.

In a more recent case reported by Alwani et al., a systematic review of sellar and parasellar brown tumors highlighted the rarity of sphenoid sinus involvement and emphasized the importance of imaging and biochemical tests for an accurate diagnosis [19]. The review also noted that surgical intervention, often combined with the medical management of hyperparathyroidism, is crucial for resolving symptoms and preventing complications.

These cases highlight the variability in clinical presentations and therapeutic approaches while emphasizing the importance of early diagnosis and multidisciplinary management. For instance, in cases where neurological symptoms are present, prompt surgical decompression is often necessary to prevent irreversible damage, as seen in the case reported by Schweitzer et al. [14]. Additionally, the role of vitamin D and calcium supplementation in managing SHPT-related brown tumors, as demonstrated in other cases, further supports the need for a comprehensive treatment strategy [15,19].

The clinical presentation of brown tumors varies depending on the anatomy of the tumor. Common symptoms include bone pain, fractures, local deformities, neurologic consequences (such as epilepsy), breathing difficulties (especially in cases involving the rib cage), functional impairment, and a reduced quality of life. The neurological symptoms are particularly dependent on the compression of adjacent neuronal structures [4,11]. For example, in the present case, the patient experienced a severe headache, intermittent nausea, diplopia, and deterioration of vision in the left eye. These symptoms are consistent with the mass effect of the tumor, which exerts pressure on nearby neural structures. It is important to note that these symptoms can also be seen in other conditions [20,21], highlighting the need for imaging and biochemical tests to aid in the diagnosis.

Biologically, elevated levels of PTH are crucial in diagnosing brown tumors, particularly when accompanied by the increased alkaline phosphate (ALP), high ionized calcium, and low phosphate levels [8]. A study by Sun et al. demonstrated that PTH assays, when correlated with several other biomarkers, can significantly narrow down the differential diagnosis [22]. Such combinations are pertinent especially in the case of an uncertain diagnosis, such as the intact PTH assay, which has become the most widely and readily available method of diagnosis, or second-generation PTH assays, which can yield falsely high PTH levels [23]. Additionally, the bone turnover marker profile is expected to improve with PTH level normalization or improvement. Furthermore, the surveillance of anti-osteoporotic medication might benefit from marker profile assessment, as seen in other types of primary and secondary osteoporosis. A supplementary contributor to high ALP is a severe vitamin D deficiency [11].

Radiologically, brown tumors present as expansive lesions with distinct imaging features. On MR imaging, they typically show low-signal-intensity masses on both T1- and T2-weighted images, with areas of increased signal intensity corresponding to hemorrhagic foci [8]. Morris et al. emphasized the importance of combining CT and MRI to accurately assess the extent of the lesion and its impact on surrounding structures, which is critical for planning surgical interventions [24]. Imaging plays a key role not only in the diagnostic process but also in monitoring the response to treatment, especially following interventions like parathyroidectomy. Recent studies suggest that [18F]-fluorocholine PET/CT may be the most effective tracer for distinguishing brown tumors from malignant tumors [11].

In cases where bone malignancy is suspected, different types of bone biopsies can aid in the management, especially for patients with a history of cancer of any origin [10,11]. However, in the case of brown tumors, the decision to perform a biopsy is individualized, given the immediate importance of obtaining a histological report (as seen in our second case). The biopsy may not always be conclusive, and the primary factors influencing the management are the treatment of the underlying parathyroid condition and the associated general and local health concerns [10,11].

The treatment of brown tumors involves addressing the underlying cause of the elevated PTH levels. In the case of PHPT, the surgical treatment of the adenoma can lead to a rapid increase in bone mineral density. Indeed, Ebina et al. documented significant bone recovery post-parathyroidectomy in the case of a giant parathyroid adenoma with multiple osteolytic fractures [25]. In SHPT, managing the underlying renal condition, along with appropriate calcium and vitamin D supplements, is crucial [4]. Furthermore, when brown tumors compress neural structures, prompt surgical decompression is strongly recommended to prevent irreversible damage, as emphasized by Ramachandraiah and Kishen [26]. The choice of the surgical approach, whether transsphenoidal or rhinotomy, depends on the size and extension of the tumor as well as the surgeon’s experience [27]. This choice is not trivial; careful preoperative planning and selection of the best route depend on various factors. These include the anatomic location of the pathologic process, and it can reduce complications and improve patient outcomes [28]. The surgical treatment of sphenoid pathology can be safely and successfully performed through a variety of approaches. The surgeon must consider several factors in selecting the optimal approach to the sphenoid sinus, including concurrent disease in other paranasal sinuses and previous sinus surgery. The anatomic location of the pathologic process, as assessed by a preoperative nasal endoscopy and radiographic imaging evaluation, can guide the surgeon in selecting the most appropriate technique [28].

The findings from our case and the literature review have several clinical implications. First, they underscore the importance of considering BTs in the differential diagnosis of lytic bone lesions, particularly in patients with hyperparathyroidism. An early diagnosis, supported by biochemical testing and advanced imaging, can prevent complications such as pathological fractures and neurological deficits. Second, the management of BTs should be tailored to the underlying cause of hyperparathyroidism. In PHPT, surgical removal of the parathyroid adenoma is often curative, while in SHPT, medical management with calcimimetics and vitamin D analogs is essential. Surgical intervention may be required for lesions causing significant compression or functional impairment. Finally, our case highlights the need for a multidisciplinary approach involving endocrinologists, radiologists, and surgeons to optimize patient outcomes.

While our case provides valuable insights, it has certain limitations. First, it is a single case report, which limits the generalizability of our findings. Larger, multicenter studies are needed to better understand the epidemiology and optimal management of BTs, particularly in rare locations like the sphenoid sinus. Second, the follow-up period in our case was relatively short. Long-term monitoring is necessary to assess the durability of treatment outcomes and the potential for recurrence. Third, the lack of histopathological confirmation in our case, while common in BT diagnosis, may raise questions about the certainty of the diagnosis. However, the combination of clinical, biochemical, and radiological findings strongly supports the diagnosis of a BT.

## 4. Conclusions

Brown tumors, though rare, should be considered in patients with hyperparathyroidism, especially when they present with unusual symptoms, including those with sphenoid sinus involvement. An early diagnosis, supported by a combination of imaging and biochemical tests, is crucial for preventing serious complications. The prompt identification allows for timely treatment and can prevent the progression of the disease. The treatment strategies should be individualized, addressing the underlying cause of hyperparathyroidism—whether primary or secondary—and the tumor’s location. In cases with neural compression or significant functional impairment, surgical intervention is necessary. Overall, a multidisciplinary approach involving endocrinologists, radiologists, and surgeons is vital to optimize patient outcomes and minimize complications.

The rarity of sphenoid sinus involvement in brown tumors underscores the importance of maintaining a high index of suspicion in patients with hyperparathyroidism who present with neurological symptoms. This case highlights the need for early intervention and a comprehensive approach to managing both the underlying endocrine disorder and its skeletal manifestations.

## Data Availability

Data are available upon reasonable request.
